# Effect of Suboptimal Neuromuscular Control on the Risk of Massive Wear in Total Knee Replacement

**DOI:** 10.1007/s10439-021-02795-y

**Published:** 2021-06-02

**Authors:** Marco Viceconti, Cristina Curreli, Francesca Bottin, Giorgio Davico

**Affiliations:** 1 Department of Industrial Engineering, Alma Mater Studiorum - University of Bologna (IT), Bologna, Italy; 2 Medical Technology Lab, IRCCS Istituto Ortopedico Rizzoli, Bologna (IT), Via di Barbiano 1/10, 40136 Bologna, Italy

**Keywords:** Finite element analyses, Wear predictions, Total knee replacements, Neuromuscular control, In silico methods

## Abstract

The optimal neuromuscular control (muscle activation strategy that minimises the consumption of metabolic energy) during level walking is very close to that which minimises the force transmitted through the joints of the lower limbs. Thus, any suboptimal control involves an overloading of the joints. Some total knee replacement patients adopt suboptimal control strategies during level walking; this is particularly true for patients with co-morbidities that cause neuromotor control degeneration, such as Parkinson’s Disease (PD). The increase of joint loading increases the risk of implant failure, as reported in one study in PD patients (5.44% of failures at 9 years follow-up). One failure mode that is directly affected by joint loading is massive wear of the prosthetic articular surface. In this study we used a validated patient-specific biomechanical model to estimate how a severely suboptimal control could increase the wear rate of total knee replacements. Whereas autopsy-retrieved implants from non-PD patients typically show average polyethylene wear of 17 mm^3^ per year, our simulations suggested that a severely suboptimal control could cause a wear rate as high as of 69 mm^3^ per year. Assuming the risk of implant failure due to massive wear increase linearly with the wear rate, a severely suboptimal control could increase the risk associated to that failure mode from 0.1% to 0.5%. Based on these results, such increase would not be not sufficient to justify alone the higher incidence rate of revision in patients affected by Parkinson’s Disease, suggesting that other failure modes may be involved.

## Introduction

A number of experimental evidences support the theory that normal healthy adults perform stereotypical, sub-maximal tasks by selecting the neuromuscular control pattern that minimises the consumption of metabolic energy.^3^ Such neuromuscular control strategy is sometime referred to as *optimal control*. By assuming optimal control, musculoskeletal (MSK) dynamics models can predict musculoarticular forces without any need to model the workings of the central nervous systems. Unfortunately, subjects affected by neuromusculoskeletal disease rarely use optimal control.^13^^–^15

The neuromuscular control one chooses to produce the same kinematics does affect the intensity of forces transmitted through the articular joints; forces transmitted at the knee can be reduced by 2 body weights (BW), depending on the neuromuscular control strategy adopted.^14^ Our recent study showed for the knee replacement patient examined in the Sixth Knee Grand Challenge,^4^ an uncontrolled manifold of 10% of the maximal muscle activation was required to account for the neuromuscular control variability observed across multiple gait cycles, which produces a variability on the resultant of the forces transmitted through the total knee replacement (TKR) also in the order of two BW or more.^13^

TKR patients adopt suboptimal control strategies primarily as compensatory patterns aimed to protect the painful knee before the operation, and also as cautionary compensation for the partial loss of proprioceptive signalling that the surgery has produced.^5^ These compensatory patterns could be attenuated using well targeted neuromuscular rehabilitation.^10^ But before exploring such approach, a more fundamental question must be asked: is this suboptimal control detrimental in any way for the patient? Suboptimal control is metabolically less efficient, but this is unlikely to be a problem for the population of interest. Thus, the only potential adverse effect might be an increased risk of implant failure due to the increased joint loading. If we look at an extreme case of suboptimal control, in patients affected by Parkinson’s Disease (PD), the answer is a clear yes: patients with PD have a much higher (5.44%) revision rate at 9 years, than the patients in the control group (1.75%).^16^ However, the very few studies on PD patients do not provide the relative incidence for each failure mode. Of all most common clinical failure modes, massive wear of the prosthetic articular surface is probably the most directly affected by joint loading,^12^ together with aseptic loosening.^6^ The aim of this work is to explore how a suboptimal neuromuscular control strategy may increase the articular wear in a metal-on-polyethylene total knee replacement, with respect to that we can expect in case of optimal control.

## Materials and Methods

### Amplification of Knee Joint Resultant Due to Suboptimal Control

A personalised MSK model of an elderly individual (male, age: 83 years, height: 1.72 m, mass: 70 kg) with a TKR, previously developed in 14 using medical imaging data from the Sixth Knee Grand Challenge,^4^ was employed in this study. Biomechanical simulations of gait were run in the OpenSim API through MATLAB (v2020b, The MathWorks Inc., Natick, MA, USA), using experimental motion capture and ground reaction force data from six overground walking trials.

While joint angles and external moments were determined *via* inverse kinematics and inverse dynamics analyses, respectively, all muscle activation patterns, muscle forces and joint contact forces at the ankle, knee and hip joints were predicted through optimization and probabilistic methods.^3^ In principle, since EMG recordings are available for some muscles, one could consider the use of EMG-driven static optimisation. But the patient examined in the Sixth Knee Grand Challenge, who shows a severely suboptimal control, does also show a considerable variability between one gait cycle and the other. Thus, rather than using EMG-driven methods, we preferred to treat the problem of suboptimal control in the frame of the uncontrolled manifold theory, assuming that any muscle activation pattern capable of producing the observed kinematics, and that would not violate the physiology limits of tetanic force for each muscle is in principle possible. The three loading conditions we considered are the one caused by optimal control (best-case scenario); the one that maximises the knee force during the gait cycle, typical of people who need to ensure the joint stability during the whole cycle (worst-case scenario), and one we obtained in a previous study, which envelops the variability of all gait cycles measured for that patient (realistic scenario).

Initially, muscle activation patterns were synthesized implementing an optimal control strategy that minimized the sum of squared muscle activations (i.e., static optimization approach 3), and the resultant forces acting on the tibia at the knee joint were extracted (JCF_act_). Then, using the uncontrolled manifold theory (i.e., probabilistic approach in Metabolica 7), a range of possible solutions (*n* = 1 × 10^5^) within a 10% variation from the optimal muscle activations was explored,^13^ to identify the largest resultant knee joint contact force profiles thus generated (JCF_met_). Finally, a suboptimal control strategy was tested, where the objective function (J_max_) was designed to maximise the knee joint contact loading (JCF_max_) while minimizing the vector of activations $$\overset{\lower0.5em\hbox{$\smash{\scriptscriptstyle\rightharpoonup}$}} {a}$$ of the muscles that did not contribute to it:

1 $$J_{{max}} \left( {\mathop a\limits^{ \rightharpoonup } ,t} \right) = w_{1} {\text{ }}\left( {\frac{1}{{\frac{{\left\| {\mathop {F^{{knee}} }\limits^{ \rightharpoonup } \left( {\mathop a\limits^{ \rightharpoonup } ,t} \right)} \right\|}}{{\left\| {\mathop {F_{{act}}^{{knee}} }\limits^{ \rightharpoonup } \left( {\mathop a\limits^{ \rightharpoonup } _{{act}} ,t} \right)} \right\|}}}}} \right) + w_{2} R\left( {\mathop a\limits^{ \rightharpoonup } ,t} \right)$$

where ${\mathop {F^{{knee}} }\limits^{ \rightharpoonup } \left( {\mathop {a}\limits^{ \rightharpoonup } ,t} \right)}$ is the resultant knee joint contact force as predicted by the musculoskeletal model, ${\mathop{F_{{act}}^{{knee}} }\limits^{ \rightharpoonup } \left( {\mathop {a}\limits_{{act}}^{ \rightharpoonup } ,t} \right)}$ is the reference knee joint contact force obtained minimizing the sum of squared muscle activations ($\mathop{a}\limits^{ \rightharpoonup }_{{act}}$), $R\left( \mathop {a}\limits^{ \rightharpoonup } ,t \right)$ is the regularization term,^14^ and *w*_1_ and *w*_2_ are constant weights defining the individual contribution of each term in the objective function. The weight ratio *w*_1_:*w*_2_ was set to 10:1.^13^ Both minimization problems were solved using the MATLAB *fmincon* function.

### Finite Element Model: Geometry and Mesh

**Figure 1:**
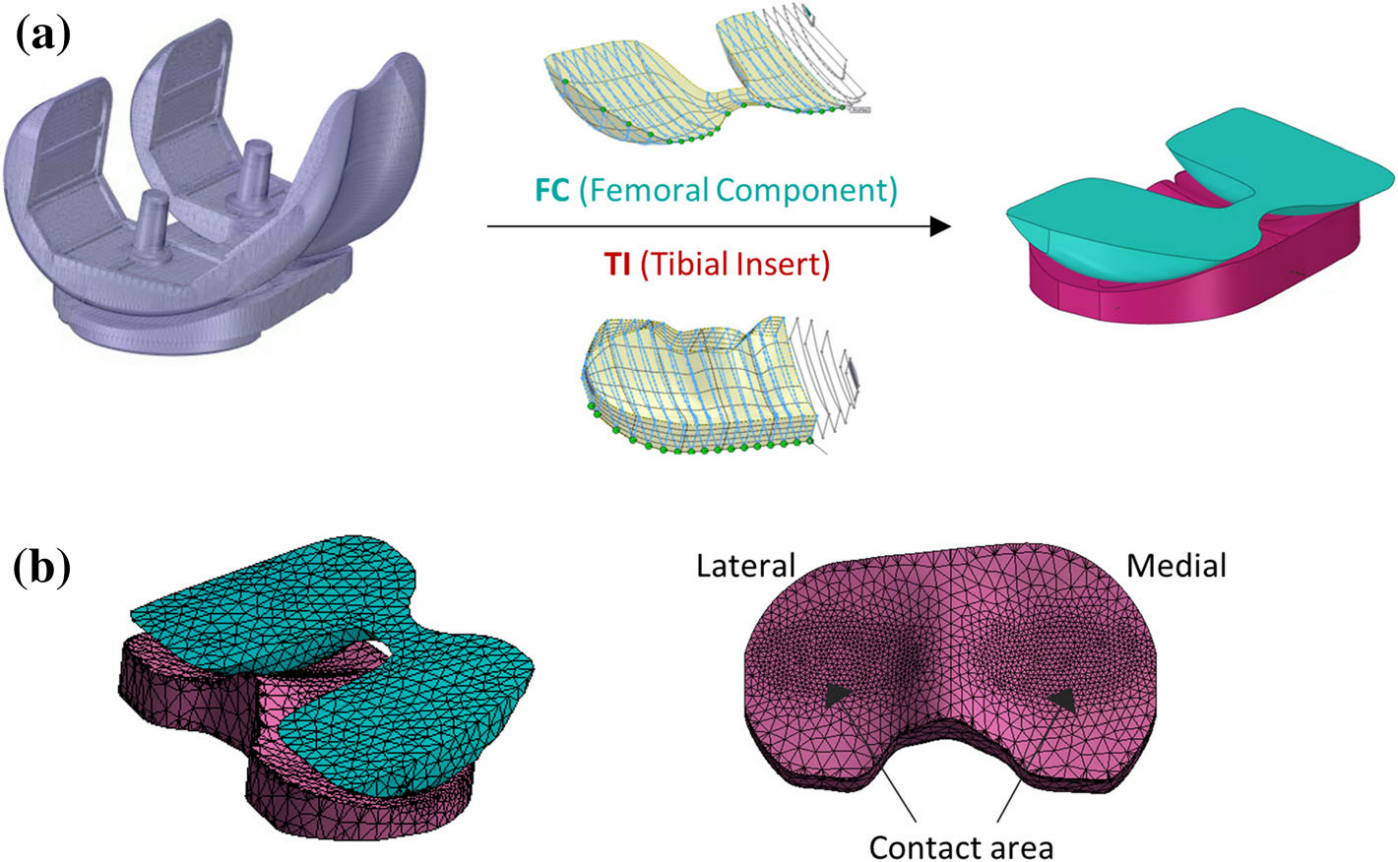
Geometry reconstruction of the tibial insert and femoral component (**a**) and mesh definition (**b**).

The three-dimensional geometry of a knee implant component (Zimmer NK-II cruciate-retaining prosthesis) 4 was reconstructed and simplified using a CAD software. Two-dimensional sketch sections were first created at the intersection of the imported surface geometry with parallel planes and then connected using the loft function in order to obtain the solid parts (Fig. 1a). A pre-processing software (Ansys Mechanical v2019, Ansys Corp, USA) was then used to develop the FE model of the TKR. The tibial insert (TI) was modelled as a linear elastic deformable material with modulus of elasticity of *E* = 463 MPa and a Poisson’s ratio (*µ)* of 0.46, while the femoral component (FC) was made of a metal alloy with significantly higher Young’s modulus (*E* = 200 GPa) and lower Poisson’s ratio (*µ *= 0.3). Both bodies were meshed with linear tetrahedral elements SOLID185. CONTACT174 and TARGET170 were used in the two contact pairs, created in order to easily analyse the wear results on the medial and lateral sides (Fig. 1b). A maximum element size of 3 mm and 1 mm was defined far from the contact and at the contact surfaces, respectively, based on a convergence study (< 1.5% difference on the total wear volume after the first 1500 cycles).

### Finite Element Model: Boundary Conditions

**Figure 2:**
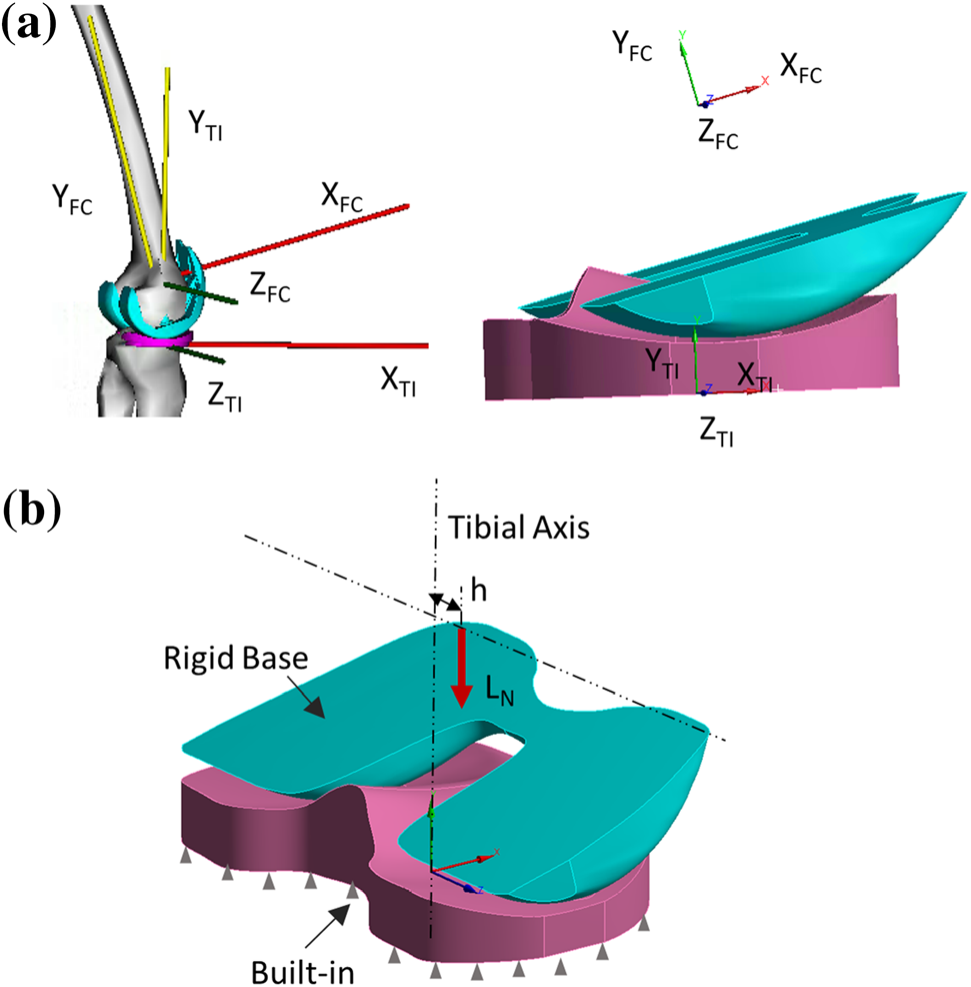
Relative position and orientation of the FC with respect to the TI (**a**) and boundary conditions (**b**) used for the FE model.

The kinematic and dynamic outputs obtained from the MSK simulations at 50% of the gait cycle (end of the stance phase) were simplified and used as boundary conditions for the FE models. Some assumptions have been made. First, the relative orientation and position of the FC with respect to the TI were defined by the flexion angle (14°) and the superior-inferior translation (45 mm), respectively (Fig. 2a). Second, the bottom surface of the TI was built-in while the axial load *L*_*N*_ (estimated by the musculoskeletal model using the three aforementioned approaches) was applied to the top rigid surface of the FC in the superior-to-inferior direction, along a line of action perpendicular to the bottom surface of the TI, with an offset *h* equal to 5 mm (6.5% of the overall width of the TI component) in the medial direction from the tibial axis (Fig. 2b).

### Contact and Wear Model

The contact was simplified as friction free using the default Augmented Lagrangian algorithm and the wear model was implemented using the TB,WEAR routine (Ansys Mechanical, v2019) with the linear version of the Archard law option. The rate of wear depth was thus computed according to the general formula:

2 $$\frac{\Delta h}{\Delta t} =k \cdot p \cdot v\quad$$

where the constant *k* is the dimensional wear coefficient, and *p* and *v* are respectively the pressure and velocity terms. Assuming the static analysis and the linear dependence of the wear rate on the sliding velocity, a fictitious wear coefficient *K*_v_ =* kv* that includes the term related to the constant and uniform sliding speed *v* at the contact nodes was introduced. The relative kinematics was thus not explicitly modelled, and the computational cost of the simulations was significantly reduced. The dimensional wear coefficient was set to *k* = 2.64 × 10^−7^ mm^3^/Nm 8 and the average sliding distance *d* per cycle was considered 25 mm with a frequency of 1 Hz.

In order to prevent mesh distortion due to the repositioning of the surface nodes when simulating material loss, the nonlinear mesh adaptivity procedure was activated with the wear-based criterion set to 0.8. Once this critical ratio of magnitude of wear to the average depth of the solid element underlying the contact elements is reached, the program automatically restarts the analysis with an improved mesh.

The material loss, attributed only to the TI, was computed during the wear simulation for a total of 3 mc. The minimum updated interval for computing the linear wear depth at each contact node as well as the volumetric wear was 1200 wear cycles.

The entire wear simulations were performed on an Intel Xeon W-2102 3 GHz workstation with 32 GB RAM. A distributed memory parallel scheme was adopted with 4 Cores.

### Simulation of the Different Neuromuscular Control Loading

The patient-specific musculoskeletal dynamics we used in this study was previously validated 13: using an optimal control the model was able to predict the measured joint loading over the gait cycle with a root mean square error of 0.5 BW (16.6% of the peak measured value). The control strategy that better tracked the measurements, presented a root mean square error of less than 0.1 BW (3.3% of the peak measured value).

The force transmitted through the implant and the sliding velocity varies during the gait cycle. However, our goal was not to pursue an accurate prediction of implant wear in a specific case (such as in 9), but rather to estimate an upper boundary for wear in case of suboptimal control. Thus, for the sake of simplicity, knowing we would err in excess, we assumed that the force and the sliding velocity were constant over the gait cycle.

On the basis of the various musculoskeletal simulations, we assumed a constant loading *L*_*N*_ of 1359 N to predict the wear in case of optimal control (JCF_act_), 2168 N for realistic suboptimal control (JCF_met_) and 10,308 N for the highest possible joint load due to suboptimal control (JCF_max_). These values represent, for each loading condition, the JCF value observed in correspondence of the second characteristic peak (i.e., 50% of the gait cycle), averaged across six trials.

## Results

### Contact and Wear Analysis

The main results obtained from the contact and wear analyses are summarized in Table 1. Generally, maximum contact pressure, contact areas, wear volume and maximum wear depth were higher on the medial side of the implant compared to the lateral side. Other typical behaviours were observed. In particular, the value of the maximum contact pressure decreased with wear due to the increased contact areas. For both the JCF_act_ and JCF_met_ configurations, the peak contact pressure in the unworn condition ranged between 13.9 MPa and 16.8 MPa on the lateral side and between 14.7 MPa and 18.2 MPa on the medial side; while for the JCF_max_ configuration, the predicted maximum contact pressure was almost three times higher. After 3 million wear cycles, these values substantially decreased (average reductions around 65%, Table 1).

**Table 1:** Maximum contact pressure, contact areas, wear volume and maximum wear depth on the tibial insert, in the unworn condition and after 3 million wear cycles

		Lateral		Medial		Total	
		Unworn condition	After 3 million wear cycles	Unworn condition	After 3 million wear cycles	Unworn condition	After 3million wear cycles
Max Contact Pressure (MPa)	JCF_act_	13.946	4.308	14.7	4.937	–	–
	JCF_met_	16.794	5.616	18.17	6.591	–	–
	JCF_max_	37.857	14.151	48.695	14.151	–	–
Contact Area (mm^2^)	JCF_act_	90.169	176.93	112.48	201.89	202.649	378.82
	JCF_met_	112.87	211.44	143.49	251.84	256.36	463.28
	JCF_max_	224.19	390.88	264.31	466.15	488.5	857.03
Volume lost (mm^3^)	JCF_act_	–	11.544	–	15.52	–	27.064
	JCF_met_	–	18.167	–	25.069	–	43.236
	JCF_max_	–	84.214		123.42		207.634
Max Wear Depth (mm)	JCF_act_	–	0.112	–	0.151	–	–
	JCF_met_	–	0.167	–	0.196	–	–
	JCF_max_	–	0.352	-	0.494	-	–

**Figure 3:**
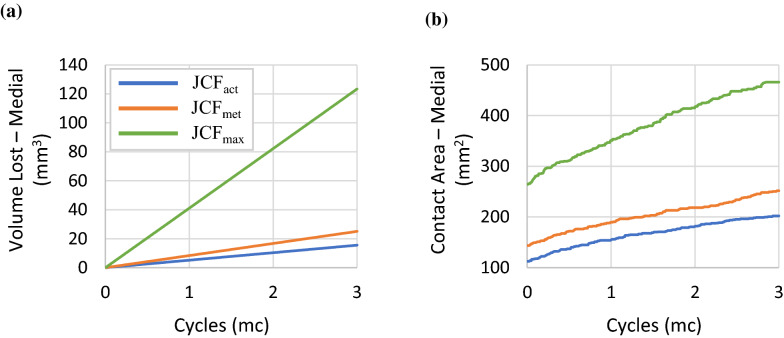
Evolution of the volume lost (**a**) and the contact area (**b**) on the medial side of the tibial implant during the wear simulation for all the three configurations (blue = optimal control, orange = 10% uncontrolled manifold, green = maximised knee joint contact forces).

The temporal trends of the increasing contact areas and volumes lost in the TI during the wear simulation are shown in Fig. 3. For simplicity, only the plots related to the medial contact side are reported. The wear volume increased linearly for all three cases reaching values of 15.52 mm^3^, 25.07 mm^3^ and 123.42 mm^3^ after 3 million wear cycles, on the medial side, respectively for configuration JCF_act_, JCF_met_ and JCF_max_. The evolution of the contact areas during wear showed a similar behaviour. The total increment in contact area on the medial side was approximately 90-108 mm^2^ for the JCF_act_ and JCF_met_ configurations, but almost twice as much (about 202 mm^2^) for the JCF_max_ configuration.

**Figure 4:**
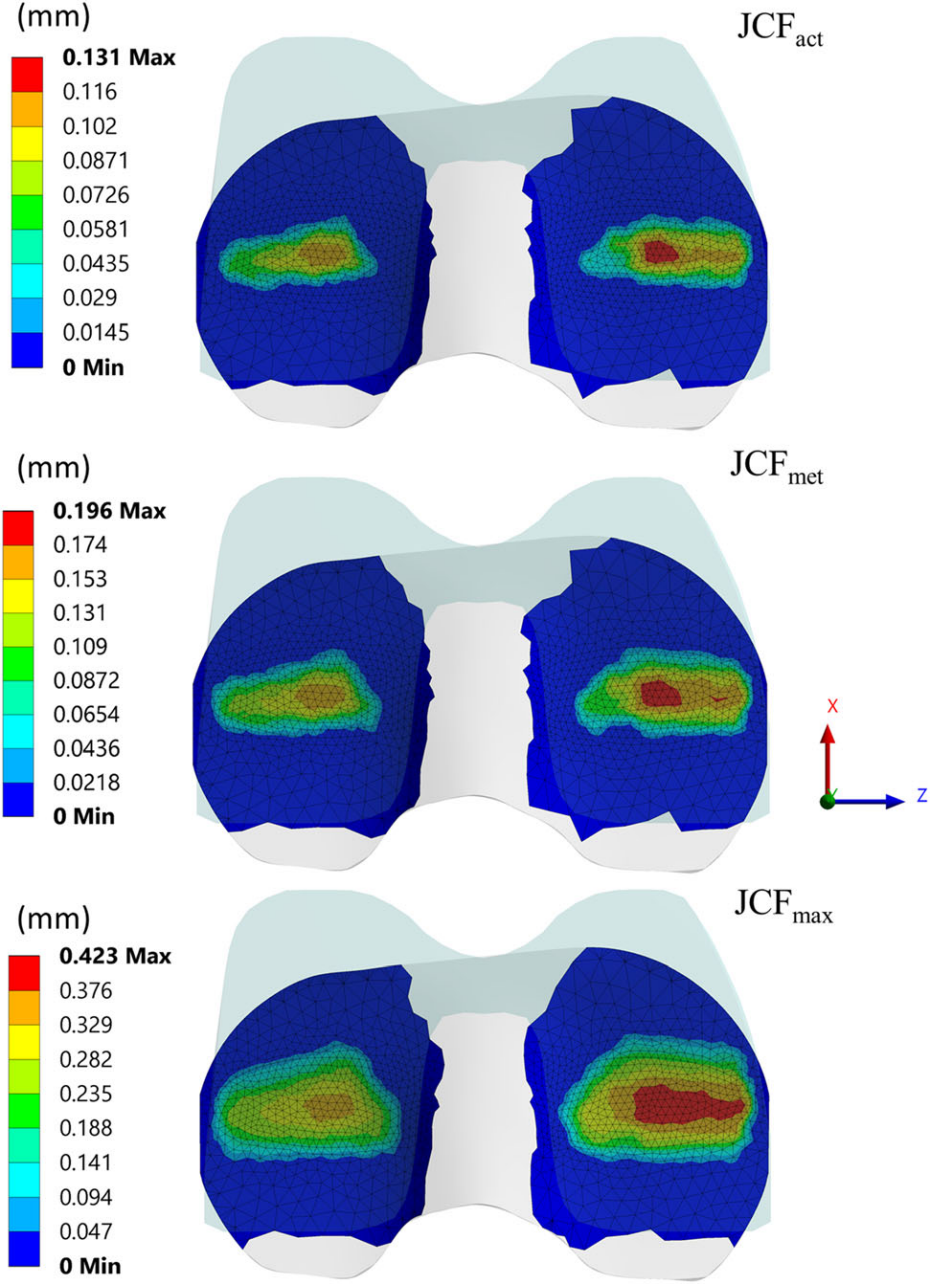
Wear maps on the tibial insert surface after 3 million cycles for the three simulated cases. Colour maps are different in each figure to maximise the contrast.

The maximum wear depth (Table 1) and the wear maps (Fig. 4) show details on wear distribution in terms of worn area location and shape. For all the simulated cases, the wear maps showed some typical features with the maximum wear depth located close to the centre of the medial side of the TI, in agreement with the loading conditions and contact surface geometries. In the JCF_max_ configuration, the wear depth and the size of the worn area were the highest and the largest overall (Fig. 4). For this specific case, the predicted maximum value of wear depth after 3 mc was 0.494 mm, about three times higher than what was found for JCF_act_ and JCF_met_ (0.151 mm and 0.196 mm respectively).

## Discussion

The aim of this work was to explore how a suboptimal neuromuscular control strategy may increase the articular wear in a metal-on-polyethylene total knee replacement, with respect to that we can expect in case of optimal control.

Because of the considerable intra-subject variability between gait cycles, we represented the effect of motor control by assuming three different peak loads in our predictions of wear: the first was predicted assuming optimal neuromuscular control (JCF_act_, *L*_*N*_ = 1359 N); the second assuming an uncontrolled manifold large enough to include all the values of joint force measured during the various gait cycles recorded (JCF_met_, *L*_*N*_ = 2168 N), and the one predicted assuming the worst possible control in term of joint loading (JCF_max_, *L*_*N*_ = 10,308 N). JCF_met_ represents the typical condition of TKR patients with mildly suboptimal neuromuscular control; JCF_act_ represents the best-case scenario, whereas JCF_max_ provides the worst-case scenario. A study on autopsy-retrieved on 20 TKRs showed that an average polyethylene wear of 17 mm^3^ per year, with a maximum of 40 mm^3^ per year.^11^ Assuming an average of one million gait cycles per year, this correlates well with the value predicted by our model (14 mm^3^) for what we consider the most realistic loading condition (JCF_met_). This figure would reduce to 9 mm^3^ per year if the patient could walk with the truly optimal neuromotor control (JCF_act_), while in the worst-case scenario the suboptimal control could cause a wear rate of 69 mm^3^ per year.

Assuming the probability of implant failure due to massive wear and osteolysis is proportional to the wear rate, a severe degradation of the neuromotor control could increase such probability of almost five folds. According to the RIPO registry 2 most recent report, the probability for a primary bi-tricompartmental TKR to fail due to tibial insert wear is around 0.1%. According to our results, a population with severe neuromotor degradation could face a risk of implant failure due to massive wear around 0.5%. Considering that, according to the same registry, the probability for a TKR patient to face a revision surgery is around 4%, and the most common cause of failure (aseptic loosening) has a risk of 1.1%, an increase of 0.4% in the risk of revision does not seem very high.

The failure rate at nine years for patients affected by Parkinson’s disease was found in a study 16 to be 5.44%, where the control group had a rate of 1.75%, a bit higher than that one reported by the RIPO registry. This means an increase of 3.69%, much larger that our model would predict (0.5%). From these results we can conclude that while a sub-optimal neuromuscular control can significantly increase the wear rate (up to 69 mm^3^ per year, in the worst-case scenario), even such increase would not justify the three-fold increase observed in the failure rate of patients with severe neuromuscular degradation. These results seem to be in good agreement with the findings of a single study on 46 TKR in PD patients, where at more than ten years of follow-up “no obvious tibial insert polyethylene wear was observed”.^1^

This study has some limitations. A first critical point regards the simplifications made when developing the wear model. As already mentioned, the TKR kinematics was not explicitly modelled, and both the force applied to the femoral component and the sliding velocity were assumed constant over the gait cycle. Also, the Archard equation used to predict linear wear does not account for the cross-shear phenomenon which is frequently introduced when dealing with joint replacement to characterize the anisotropic material behaviour of the plastic component.

Additionally, it is important to underline that the only effect of the different loading conditions was considered when comparing the three configurations (JCF_act_, JCF_met_ and JCF_max_) in terms of wear predictions. One fundamental assumption of the study was in fact that the three neuromuscular control strategies produced the same kinematics.

Despite these limitations, that are worth to be considered when an accurate TKR wear prediction is needed, the results here presented provide an upper boundary to increase in massive wear one can expect from suboptimal control. Given that even this probably overestimated effect does not justify the increase in failure rate observed in patients with severe neurodegenerative conditions, this work call for a similar exploration for the other potential candidate, aseptic loosening.
